# Supplementary material for: Monkeys can identify pictures from words
Source: PLoS One. 2025 Feb 12;20(2):e0317183. doi: 10.1371/journal.pone.0317183 (PMC11819547; doi:10.1371/journal.pone.0317183)
Supplement: S1 Table — (PDF) [file pone.0317183.s002.pdf]

S1 Table. Monkeys' learning parameters and hit rate.

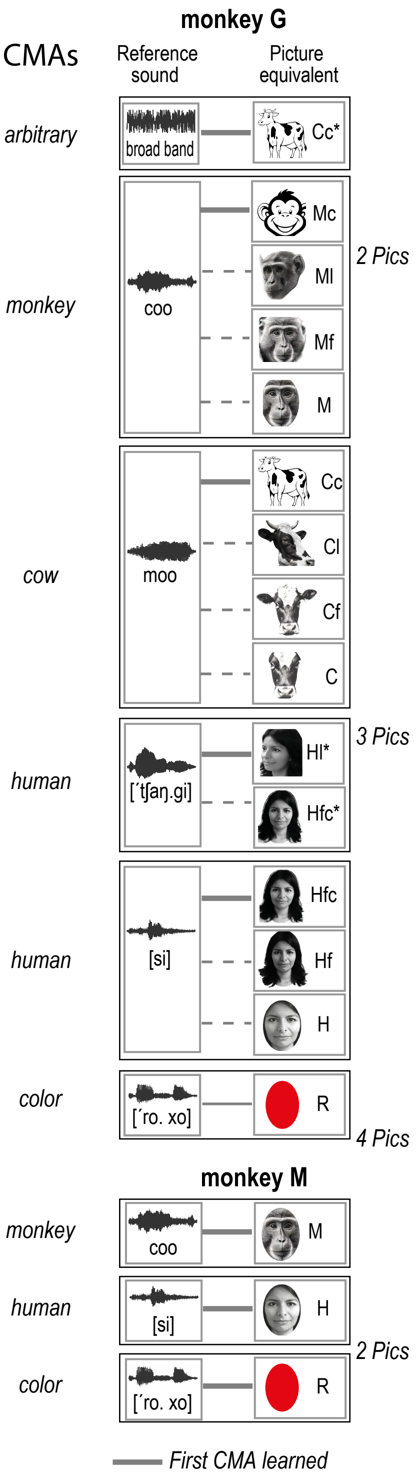

CMA sets in a session (parameters correspond the correct match shown in bold)	HR at y0 (%)	Sessions to gamma	Sessions to delta	HR (%) at delta (mean ± SD)	Sessions to lambda	HR at lambda (%)
**Mc**, Cc*	47	8	15	71.30 ± 10.08	28	82
Mc, **Cc***	55	4	1	65.61 ± 12.74	42	67
**Mc**, Cc	99	1	3	71.37 ± 7.65	30	74
Mc, **Cc**	29	4	8	72.13 ± 10.95	25	73
**Mc**, Cc, Hl*	114	1	4	72.84 ± 11.7	94	73
Mc, **Cc**, Hl*	133	1	7	73.73 ± 12.78	119	74
Mc, Cc, **Hl***	5	4	4	70.63 ± 11.98	122	71
**Mc**, Cc, Hfc*	80	1	1	80.83 ± 8.0	72	81
Mc, **Cc**, Hfc*	91	1	2	74.61 ± 6.61	71	74
Mc, Cc, **Hfc***	76	1	2	71.74 ± 5.88	71	71
**Mc**, Cc, Hfc	29	3	5	82.94 ± 8.67	10	79
Mc, **Cc**, Hfc	16	3	5	80.44 ± 8.03	10	84
Mc, Cc, **Hfc**	49	2	2	73.16 ± 1.02	13	73
**Mc**, Cc, Hfc	52	1	2	70.89 ± 17.45	63	73
Mc, **Cc**, Hfc	76	1	2	79.59 ± 9.95	105	80
Mc, Cc, **Hfc**	66	1	3	80.83 ± 8.74	104	81
**Ml**, Cc, Hfc	59	1	4	82.27 ± 6.65	149	82
Ml, **Cc**, Hfc	64	1	3	77.35 ± 7.48	150	77
Ml, Cc, **Hfc**	108	1	2	86.77 ± 7.85	146	87
**Ml**, Cl, Hfc	66	1	4	85.23 ± 8.35	76	85
Ml, **Cl**, Hfc	41	1	4	81.6 ± 7.48	76	80
Ml, Cl, **Hfc**	105	1	4	85.51 ± 9.18	76	85
**Mf**, Cf, Hfc	57	1	10	89.07 ± 5.58	24	90
Mf, **Cf**, Hfc	50	1	9	86.23 ± 5.79	25	88
Mf, Cf, **Hfc**	57	1	5	88.41 ± 12.32	29	88
**Mf**, Cf, Hf	66	1	6	88.1 ± 1.62	8	86
Mf, **Cf**, Hf	75	1	0	82.95 ± 5.37	13	167
Mf, Cf, **Hf**	97	1	2	90.68 ± 4.59	12	93
**M**, C, H	92	1	1	91.04 ± 3.91	31	92
M, **C**, H	106	1	2	87.29 ± 4.26	30	87
M, C, **H**	109	1	2	86.85 ± 5.07	30	88
**M**, C, H, R	89	1	6	74.2 ± 10.98	56	74
M, **C**, H, R	49	1	5	75.05 ± 10.51	57	75
M, C, **H**, R	116	1	5	88.10 ± 8.26	57	89
M, C, H, **R**	43	1	14	70.46 ± 12.92	48	82
**M**, C, H, R	73	1	3	86.05 ± 8.98	208	86
M, **C**, H, R	65	1	4	83.33 ± 7.91	207	84
M, C, **H**, R	105	1	4	88.15 ± 9.52	207	88
M, C, H, **R**	40	1	3	74.83 ± 10.37	208	74
**M2**, H	75	1	7	91.75 ± 3.18	6	98
M2, **H**	89	1	2	86.42 ± 7.17	11	88
**M2**, R	79	1	2	84.95 ± 3.29	9	84
M2, **R**	93	1	2	82.33 ± 3.68	9	87
