# Supplementary material for: Monkeys can identify pictures from words
Source: PLoS One. 2025 Feb 12;20(2):e0317183. doi: 10.1371/journal.pone.0317183 (PMC11819547; doi:10.1371/journal.pone.0317183)
Supplement: S2 Table — (PDF) [file pone.0317183.s003.pdf]

S2 Table. Overall hit rate (mean ± STD) in four CMAs

Number of Pictures discriminated on the screen	CMAs 'monkey' (coo / monkey face)	CMAs 'cow' (moo / cow face)	CMAs 'human' ([si] / human face)	CMA 'color' ([ˈro.xo] / red oval)
	**monkey G**			
2 pictures	75.35 ± 10.70	74.14 ± 10.80	73.16 ± 10.29	…
3 pictures	82.24 ± 11.32	80.24 ± 8.47	85.20 ± 8.92	…
4 pictures	87.43 ± 8.17	85.19 ± 7.71	90.17 ± 6.39	77.70 ± 8.94
	**monkey M**			
2 pictures	88.95 ± 4.76	…	87.08 ± 7.20	82.93 ± 3.95
