# Supplementary material for: Monkeys can identify pictures from words
Source: PLoS One. 2025 Feb 12;20(2):e0317183. doi: 10.1371/journal.pone.0317183 (PMC11819547; doi:10.1371/journal.pone.0317183)
Supplement: S3 Table — Selections of pictures during hits and FAs in the condition when one sound was associated with different pictures of the same type. (PDF) [file pone.0317183.s004.pdf]

S3 Table. The proportion (mean ± STD) of pictures selected

Sound	Associated pictures	Selected picture (%)		
		monkey	cow	human
coo	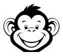 Mc	**68.1 ± 18.81**	21.79 ± 5.95	10.11 ± 16.72
	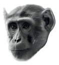 Ml	**84.85 ± 8.52**	12.62 ± 6.6	2.53 ± 2.92
	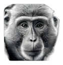 Mf	**83.97 ± 13.79**	13.33 ± 14.24	2.71 ± 3.79
	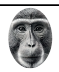 M	**91.05 ± 3.98**	6.23 ± 3.6	2.73 ± 1.78
moo	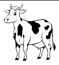 Cc	14.86 ± 13.32	**79.16 ± 10.41**	5.99 ± 4.99
	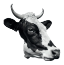 Cl	14.53 ± 6.59	**81.18 ± 7.95**	4.29 ± 3.1
	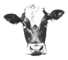 Cf	14.37 ± 12.02	**81.88 ± 11.76**	3.76 ± 3.58
	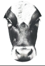 C	9.82 ± 3.56	**87.24 ± 4.28**	2.95 ± 2.27
[si]	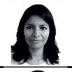 Hfc	5.85 ± 7.36	11.18 ± 6.59	**82.97 ± 8.67**
	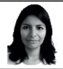 HFc1	5.15 ± 4.45	9 ± 5.48	**85.85 ± 9.24**
	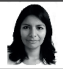 Hfc2	4.45 ± 5.13	6.86 ± 7.24	**88.69 ± 11.76**
	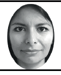 H	4.37 ± 2.5	8.67 ± 3.96	**86.96 ± 5.02**
