# Supplementary material for: Monkeys can identify pictures from words
Source: PLoS One. 2025 Feb 12;20(2):e0317183. doi: 10.1371/journal.pone.0317183 (PMC11819547; doi:10.1371/journal.pone.0317183)
Supplement: S4 Table — (PDF) [file pone.0317183.s005.pdf]

S4 Table. Hit rate (mean ± STD) in different versions of the learned sounds.

**Monkey G**	coo	moo	[´ro.xo]	[si]
(L)earned	91.4 ± 16.98	84.59 ± 17.17	70.34 ± 23.46	89.38 ± 19.73
S1	73.59 ± 22.31	75.99 ± 19.91	63.92 ± 21.21	89.38 ± 19.51
S2	64.23 ± 23.17	79.3 ± 19.22	62.84 ± 22.19	91.18 ± 19.34
S3	55.79 ± 26.38	72.96 ± 19.84	62.62 ± 21.09	90.42 ± 19.69
S4	70.07 ± 23.4	77.83 ± 19.9	65.91 ± 19.34	91.2 ± 19.77
S5	80.98 ± 21.23	77.58 ± 17.52	72.23 ± 22.33	74.86 ± 24.72
S6	82.97 ± 20.29	70.16 ± 20.97	72.52 ± 19.97	71.94 ± 24.87
S7	89.27 ± 17.21	52.47 ± 21.19	69.89 ± 21.94	83.15 ± 22.23
S8	56.84 ± 25.24	73.6 ± 21.32	63.62 ± 22.86	79.3 ± 21.7
S9	24.18 ± 19.36	62.95 ± 21.22	52.13 ± 24.95	90.86 ± 19.44
S10	78.17 ± 22.87	88.37 ± 15.69	65.95 ± 21.59	88.77 ± 21.64
o-o	81.29 ± 9.15	87.91 ± 7.9	82.04 ± 8.88	82.51 ± 9.15
S1-S1	79.35 ± 10.73	87.2 ± 7.29	82.23 ± 8.27	81.09 ± 9.15
S2-S2	77.5 ± 11.46	81.04 ± 11.45	86.06 ± 7.64	80.49 ± 10.39
S3-S3	79.45 ± 9.65	85.09 ± 10.29	82.81 ± 10.07	81.45 ± 9.05

**Monkey M**	coo	[´ro.xo]	[si]
(L)earned	92.22 ± 5.91	85.83 ± 11.5	88.08 ± 12.18
S1	74.01 ± 19.01	77.05 ± 16.61	72.78 ± 21.95
S2	87.83 ± 11.91	57.5 ± 18.12	60 ± 25.5
S3	78.45 ± 17.01	87.21 ± 11.4	56.11 ± 21.76
S4	83.81 ± 13.02	86.25 ± 8.67	65.99 ± 25.59
S5	70.24 ± 19.87	47.5 ± 16.47	67.22 ± 19.64
S6	88.75 ± 9.91		
