# Supplementary figures and images for: Monkeys can identify pictures from words
Source: PLoS One. 2025 Feb 12;20(2):e0317183. doi: 10.1371/journal.pone.0317183 (PMC11819547; doi:10.1371/journal.pone.0317183)

S1 Fig. Learning of CMAs

2 pictures

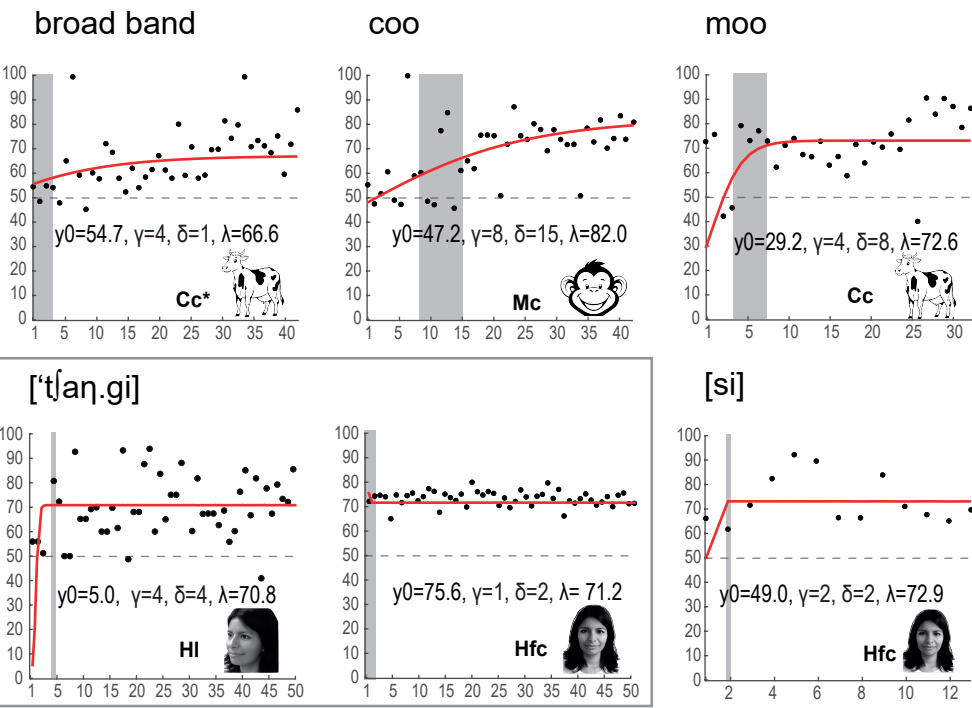

3 pictures

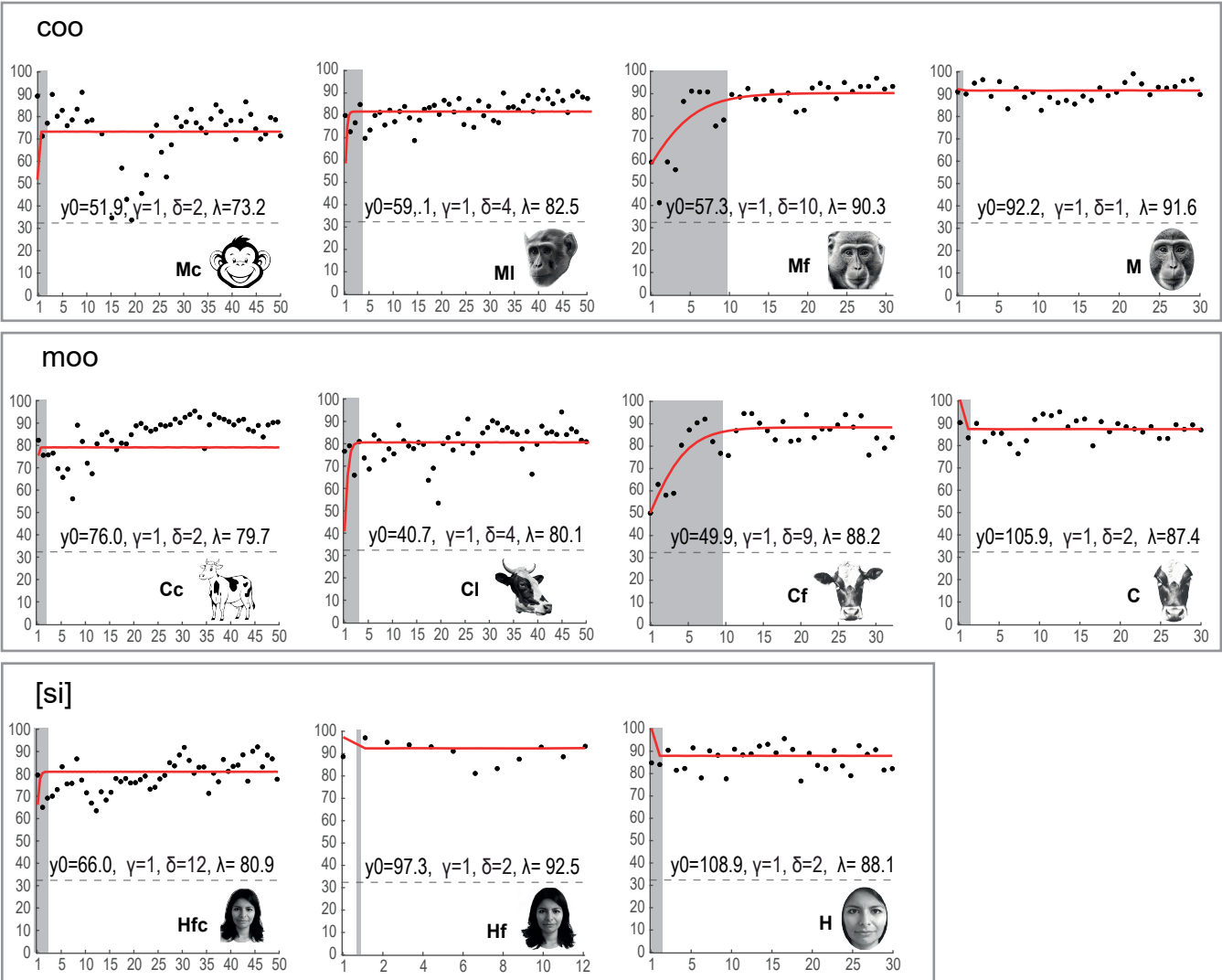

4 pictures

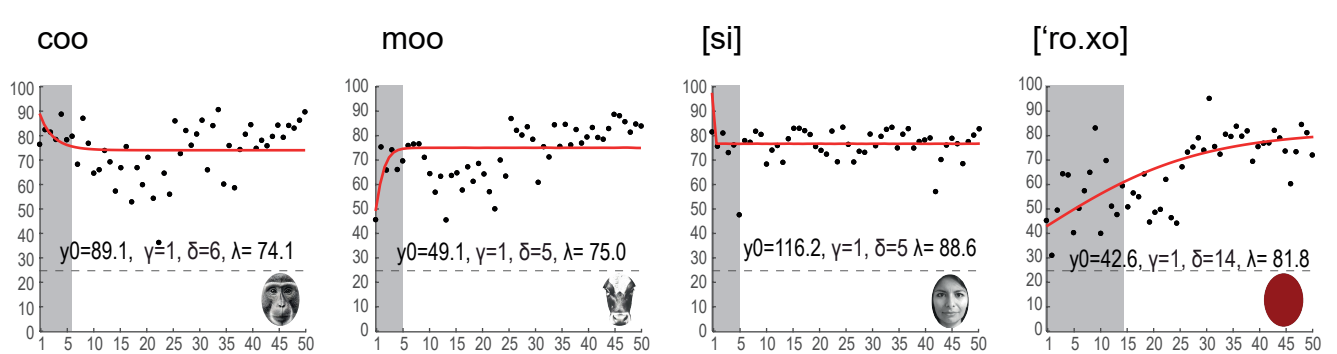

Supplement: S1 Fig — (PDF) [file pone.0317183.s006.pdf]

**S2 Fig. Hit rate and reaction times at different picture locations**

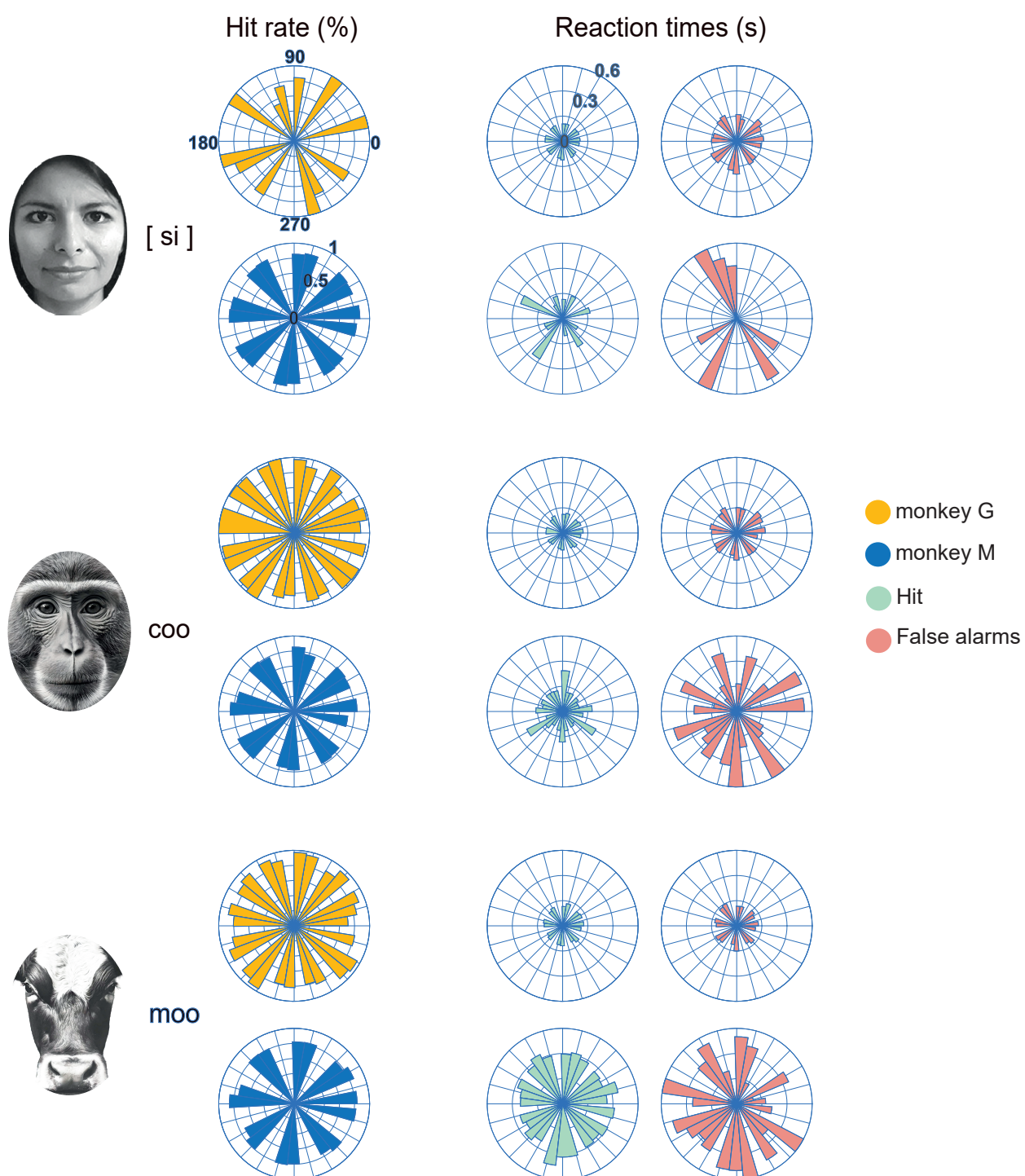

Supplement: S2 Fig — To analyze biases toward selecting a P at any angle from the center of the touchscreen, we performed a one-way ANOVA, False Discovery Rate corrected for multiple pairwise comparisons. Monkey M showed no location bias (p-values > 0.034). Monkey G, however, exhibited a significant effect for the monkey face position (F [15, 160.67] = 1.97; p = 0.014) and the cow face (F [15, 150.619] = 2.51; p = 0.001), but not for the human (p = 0.988). Post-hoc analysis (Tukey’s HSD) revealed these differences occurred in angles < 90° within each screen quadrant. In other words, while there were biases in selecting pictures at angles, there was no consistent preference for a specific quadrant. Based on these findings, the behavioral results presented here correspond to subsequent experiments presenting pictures only in four quadrants. (PDF) [file pone.0317183.s007.pdf]
