# Supplementary material for: Monkeys can identify pictures from words
Source: PLoS One. 2025 Feb 12;20(2):e0317183. doi: 10.1371/journal.pone.0317183 (PMC11819547; doi:10.1371/journal.pone.0317183)
Supplement: Attachment — Submitted filename: Response to Reviewers.docx [file pone.0317183.s008.docx]

**Responses to the reviewers (**highlighted in blue)

**Reviewer #1:**

The manuscript of Cabrera-Ruiz is focused on macaques‘ abilities to learn audio-visual crossmodal associations in a delayed match-to-sample task.

Although the text is generally very well written, in my opinion the main focus/emphasis of the paper is not stated clearly enough. As far as I understand, one of the main achievements of the study is the new procedure for training macaques to perform the audio-visual cross modal task. However instead of emphasizing that achievement and discussing it properly, the authors extensively comment on (arbitrary) proposed models of cross-modal associations, which cannot be tested without application of electrophysiological methods.

The revised manuscript now emphasizes the achievement of training macaques in cross-modal discrimination. For example, line 8 of the **Abstract** now reads: *"We found that the monkeys learned and performed proficiently in over a dozen associations."* We also included full descriptions of the training protocol as a ***"Monkeys training"*** subsection at the Methods (lines 146-177), and a ***"Learning measurements"*** subsection about the analytical methods for evaluating the learning process (lines 178-203). In addition, we included a new section *"****Rhesus Monkeys Can Learn Cross-Modal Associations Between Stimuli of Different Types****"* at the Results (lines 251-294), and included S2 Fig in the text as Fig 2 (lines 277-282) with better descriptions of the learning of CMAs across training sessions, and a new panel at Fig 2B (also suggested by Reviewer 2), showing the number of sessions to performing each CMA above chance. Finally, we created a new S1 Fig showing the performance for each CMA across sessions.

We also agree with the reviewers about the problem of presenting models about the neuronal mechanisms of CMAs without evidence. Therefore, we decided to remove Figure Four from the document, limiting ourselves exclusively to adding information about cross-modal association models in the introduction. We now refer to the acoustic template model as a mechanism for association between modalities and to the alternative model of learning through social interactions. These models are important for the association between modalities as a basis for associations between stimuli that are not necessarily ethological but that are used in humans for language (which was what we tried to test in our study). The first paragraph of the introduction now reads as follows:

“*Humans form cross-modal associations (CMAs) between sounds and images, which play a vital role in integrating semantic representations within language [1]. Supporting this, fMRI studies have shown that the temporal lobe of the human brain is actively involved in CMAs [2,3] between words and visual objects [4]. It is believed that CMAs between phonological "templates"—developed in human infants by listening to caretakers—and observed objects are essential for creating semantic representations and aiding the production of a child's first words [5–8]. Similarly, auditory templates have been proposed as a mechanism for vocal production in birds [9–13] and marmoset monkeys [14]. Recent studies, such as those by Carouso-Peck and Goldstein [15,16], have also shown that visual signals during social interactions can also influence vocal production in birds. However, only a few ethological studies have suggested the existence of CMAs between vocal sounds and visual cues for semantic communication [17]. For instance, research has observed that vervet monkeys respond to calls signaling the presence of predators by looking upwards, downwards, or climbing into trees [18].*”

Major comments:

Introduction: More information (including existing models) about cross-modal association in macaques should be provided.

We included important references to cross-modal association processes in monkeys. Importantly, we also framed the introduction within the scope of working memory since it plays a key role in multisensory association processes (as noted by reviewer 2). The second paragraph of the introduction now reads as follows:

*“Neurophysiological recordings in monkeys have shown that the prefrontal cortex (PFC) — a brain area homologous to that in humans — utilizes working memory (WM) circuits [19] to perform CMAs between voices and faces [20–32], receiving inputs from various sensory regions [33–36]. CMAs have also been observed in the auditory and visual areas of the temporal lobe [37–45]. Notably, trained macaques have demonstrated the ability to perform cross-modal discriminations between visual and tactile objects [46,47], and between stimuli that could be considered non-ethologically relevant (NER), such as between pitch and color [48] and between amodal information (i.e., information that does not belong to a particular modality) [49] such as numerosity [50] and flutter frequencies [51–54]. However, it remains to be explored whether non-human primates can establish CMAs between NER stimuli that are important for human language, like words —which monkeys can discriminate phonetically [55,56], and pictures.”*

Line 42: The authors mention that the subjects are relatively old, which makes me think that they have experience in many other tasks. It would be good to comment whether and in what way the previous tasks, which the subjects were trained in, could affect their ability to learn the crossmodal associations.

The monkeys had not participated in any experiments previously nor had they been trained in any task. We now clarify this in the Methods on line 69, which now reads as follows:

*“The animals had no previous training in any other task and were not subjected to any surgery or head restraint for this behavioral study.”*

Line 49: What would be the influence of the environmental enrichment to the performance of the audio-visual task?

Environmental enrichment was implemented as part of the animals' housing, e.g., plastic boxes with snacks inside to promote manual dexterity. Additionally, the monkeys spent time in a shared space with room to climb and socialize with other monkeys, which apart from favoring the display of social behaviors characteristic of their species, probably played a role in the 'monkey' CMA. Similarly, the animals were partially exposed to the faces of the researchers (i.e., they wore masks), and only the picture of the authors was used as a stimulus. Therefore, we believe that it is unlikely it helped the monkeys significantly in creating the associations for the task. The lines 77-82 address enrichment as follows:

“*The monkeys also had access to an enriched environment with toys, a recreation area for climbing and socializing with other monkeys four days a week, and opportunities for grooming through mesh sliding doors. In addition, cartoons and wildlife videos of content unrelated to the experiments, were presented on TV for no more than four hours a day. However, the face and voice of one of the researchers with whom the monkeys interacted were used during the experiments”.* In this regard, line 393 of the discussion reads: *“We found no clear evidence that learning CMAs that included possible ethologically relevant stimuli like human and monkey faces, or coos [20–31] were facilitated more than other CMAs to which they had no previous exposure.”*

Line 94-102 – Monkey Training: In my opinion this is one of the most important methodological part of the paper. More details should be provided regarding the training procedure.

We expanded the Methods section ("*Monkeys Training,"* lines 146-177) to include a detailed description of the training protocol in the Methods section ("*Learning Measurements*," lines 178-203), where we describe analytical methods of the monkeys’ learning process. We incorporated S2 Fig as Fig 2 so the monkeys' learning of CMAs can now be observed—a critical finding in our study. We also included S1 Fig to show the performance at all CMAs throughout learning and added sections to the Results and the Discussion specifically addressing the learning of CMAs. Specifically, sections *"Rhesus Monkeys Can Learn Cross-modal Associations Between Stimuli of Different Types"* (lines 251-294), and *"Rhesus Macaques Create Crossmodal Associations Between Sounds and Images of Different Types"* (lines 377-417), respectively.

Line 146 / Fig 2C, insets: It would be interesting not just to present the FA rates, but also to show which are the most common mistakes (e.g. are monkey faces confused more often with human faces in comparison to the red oval).

Addressing this question, we included pie charts in Figure 3 (formerly Figure 2), showing the pictures selected during false alarms. We also included better descriptions of the results (lines 316-331) and a new S3 Table with the percentage of choices during hits and false alarms.

Lines 188-195: The authors identify 3 key findings, the first 2 of which directly follow the presented results. The third finding claims that “the monkeys’ success in forming crossmodal associations required […] working memory”, which is logical, however it is unclear how it could be derived from the results.

Regarding working memory, our task required it due to the 3s delay between auditory stimuli and picture options. We now provide better descriptions in the abstract, introduction, methods, results, and discussion. The abstract (lines 6-8) now reads: “In each trial, the monkeys listened to a brief sound (e.g., a monkey vocalization or a human word), and retained information about the sound to match it with one of 2–4 pictures presented on a touchscreen after a 3-second delay.” The Introduction (lines 45-50) now reads: “We specifically designed the task to temporally separate auditory and visual stimuli, thus engaging WM circuits to retain one modality in mind while awaiting the corresponding cross-modal stimulus. Unlike prior studies, this task required the monkeys to retain auditory information during a 3-second WM period and then use this information to select a matching visual from a set of 2-4 pictures displayed simultaneously on a screen after the delay.” The Methods (lines 113-114) read: *“We trained two rhesus macaques in a DCMMS task to assess their ability to establish CMAs between sounds and images temporally decoupled.”* The Results (lines 219-221) read: *“Each trial commenced with the monkeys hearing a reference sound, followed by a 3-second delay, after which 2-4 pictures were displayed on the screen.”* Finally, the Discussion mentions that the type of information retained in WM was information evoked by the sound, reading as follows: *“To solve the task, monkeys had to retain in WM either an auditory replay or a crossmodal equivalent of the sounds (i.e., a face) and compare the memory against different pictures to find the match.”* (lines 367-369)

Lines 208-212: In my opinion this is the most important contribution of the study and I think that it should be discussed more extensively.

We included a new section in the discussion (lines 377-417) called *"****Rhesus Macaques Create Crossmodal Associations Between Sounds and Images of Different Types,****"* in which we discuss our results based on the points raised in the discussion about the ability of monkeys to make cross-modal associations, and based on the learning models presented in the instruction. The section reads as follows:

“*Previous studies demonstrated that monkeys could perform crossmodal discriminations of supramodal information such as numerosity and flutter frequencies [50–54] and learn and group numerous sounds into categories irrelevant to their ethology [55,56]. However, establishing cross-modal associations between NER categories in monkeys has proved to be challenging [37,46–48]. In training two rhesus monkeys in the DCMMS task, we initially encountered hurdles as the monkeys tended to disregard sounds [64,65]. To counter this, training began with sound detection and progressively moved to crossmodal associations. We obtained different learning parameters from the monkeys’ performances in each CMA across sessions (****Fig 2****).*

*During the initial training phase, the monkeys learned to interact with the task’s apparatus (i.e., pressing the lever and touching the screen), achieving controlled motor responses within one or two weeks. Learning the first CMA (i.e., a broadband noise paired with a cow cartoon) required many sessions. Subsequent CMAs achieved statistical performance in just a few sessions; however, the animals excelled at the task after many practice sessions. We found no clear evidence that learning CMAs that included possible ethologically relevant stimuli like human and monkey faces, or coos [20–31] were facilitated more than other CMAs to which they had no previous exposure. In other words, the animals learned all CMAs at similar rates, providing behavioral data that could be highly informative regarding the brain responses underlying CMAs. Future neurophysiological evidence could build on these behavioral findings.*

*Three of our results aligned with the idea that CMAs could be created from templates [5–12]: 1) monkeys learn each new CMA faster; 2) mastering a CMA requires a prolonged period, akin to learning to speak in humans; 3) the animals' performance remained consistently high when the same vocalizations or words were presented with different voices, suggesting that the acoustic variations activated auditory templates, similar to how formants in words trigger acoustic recognition in monkeys [55]. Similarly, our results suggest that visual templates could create perceptual equivalence among different faces of the same type (****Fig 3B****). This is the strongest evidence to date that supports the possibility that monkeys can connect auditory and visual templates as humans do.*

*The formation of supramodal circuits linking vocalizations with other motor behaviors [12,13] has suggested that the integration process in NHPs might similarly involve motor and spatial associations across sensory modalities [66,67]. In our task, such associations were unnecessary since the animals had to match a sound with the corresponding picture, which was presented at different locations every trial. Moreover, studies exploring the convergence of crossmodal information in WM [22–27,48] indicate that while motor or spatial associations may facilitate initial learning, more abstract associations such as numerosity or flutter [50,54], extending beyond immediate and innate categories, can be developed through direct CMAs. Therefore, the monkeys performing our task could have created direct connections between auditory and visual templates.”*

Lines 232-270: The two presented models are very interesting, however in the context of this behavioral experiment they are completely arbitrary. I am not sure whether they should be discussed so extensively without any electrophysiological data which can support either of them.

As we said before, we only mention in the Introduction and Discussion what is known about crossmodal associations from electrophysiological experiments in monkeys. In addition, following the suggestion of both reviewers, we decided to remove Figure 4, which presented possible neural mechanisms for making crossmodal associations, limiting ourselves exclusively to presenting the possibilities in lines 426-434 of the discussion, reading: *“A candidate brain region for the type of WM involved in our task is the PFC [19], which participates in the retaining of parametric and nonparametric information of different sensory modalities compared intra- or cross-modally [2,3,20–31]. Notably, the PFC is also responsible for intramodal associations of stimuli separated in space and time [50]. Therefore, it is probably capable of translating information cross-modally; in our task, this could involve possibly invoking visual representations after hearing sounds, thus retaining visual information in WM for later comparisons with the pictures, rather than keeping the reference sound in working memory until the pictures appear.”*

Minor comments:

Line 2: the sentence starting with “To find out” is ambiguous. To find out what?

We rephrased the Abstract to: *"Humans learn and incorporate cross-modal associations between auditory and visual objects (e.g., between a spoken word and a picture) into language. However, whether nonhuman primates can learn cross-modal associations between words and pictures remains uncertain."*

Line 98: probably “cue to” instead of “clue for”.

The description of the task was improved. We now explain that such a cue was a cross appearing at the center of the touchscreen to indicate the beginning of the trial. The descriptions appear in the Methods at lines 116-117, reading: *"Each trial began with a white 1° cross appearing in the center of the touchscreen."*

**Reviewer #2:**

Review for “Crossmodal association between auditory and visual objects in rhesus monkeys” PONE-D-24-30219. This paper assessed whether rhesus macaques can learn crossmodal associations, how they are perceiving the visual sets versus the auditory sets in those associations, and what models of visual or auditory memory might explain these patterns. They successfully trained two macaques on a set of crossmodal associations (CMAs), which is no trivial feat. Performance on the task is well presented and clearly above chance. Upon further assessment, macaques appear to treat different individual sounds of the same type as perceptually equivalent, whereas patterns of reaction times for different images suggests this might not be the case for the visual image sets. This has interesting implications for how macaques may form these types of representations, and the authors put forth some possible models for how these representations might be stored in memory to explain these patterns.

I believe this experiment is interesting and relevant to ongoing work in the field. The data is compelling, and the experimental design effectively assesses their questions. There is room for improvement in how the paper is framed, as well as in the clarity of some of the explanations and data presentation, but I believe the work itself is sound, and will be useful groundwork for future studies in the field. I recommend this paper for publication, pending some revisions based on my suggestions below.

General Suggestions

1) Framing of memory systems in the intro. Your intro only talks about assessing whether macaques can learn CMAs. This makes sense since that’s a major point of your study, but this paper isn’t really about the CMA learning itself. I spent most of the paper thinking about the learning and acquisition of CMAs, since that’s how it’s framed here, but you don’t really make any points about acquisition at all. So much of your discussion is dedicated to how those CMAs are represented and maintained in the brain, and using macaques as a model system for assessing these different possible neural models. You need to lay the groundwork for that here. Are there existing competing hypotheses about how CMAs are represented in humans or macaques that you could discuss here? At the very least you need to mention that CMAs can be held in working memory, or that memory is relevant to this process at all. Even some basic background about audio versus visual working memory and how they might be relevant here would help readers keep that framing in mind and not be blindsided by the discussion.

As suggested by the reviewer, we have given a different context to the paper, specifically using working memory and how cross-modal associations depend on it as a context and presenting models of cross-modal association learning. Thus, the changes in the Abstract, Introduction, and Discussion are significant. While the results did not change, they now include descriptions of the learning process (Fig 2 and S1 Fig). In addition, we added a section in the Discussion, *"****Working Memory Mechanisms for Crossmodal Matching,****"* lines (418-447), that explicitly addresses how WM relates to cross-modal association processes as in our task.

2) How you’re referring to CMAs and sets of CMAs. It took me a while to figure out how exactly your different, specific CMAs were grouped (i.e. one single coo was trained to be associated with a few different monkey images, and each of those individual associations was one CMA). It would be useful to clarify this early, and possibly give name to the different CMA type groups (i.e. CMA sets or something like that), because knowing the associations were one coo to multiple images changes how a reader might interpret the results. I know you added more coos later on, but I spent the majority of the paper thinking each CMA was made up of completely different individual sounds and images. Even the S3 table didn’t clarify this for me, as I read the list as being grouped into categories (i.e. “coos”) with the individual pictures presented, and I interpreted the letters next to each picture as specifying the name of the specific sound used with that picture, rather than as the identifying name of the picture itself.

We were careful to explain what CMAs consist of in the Methods section *"****Delayed Crossmodal Match-to-Sample Task****,"* lines 125-131, which read as follows: *"Each CMA was established by associating a sound with a picture representing the same category of external stimulus (e.g., both corresponding to a human). For example, a CMA of the type 'human' consisted of the association between the word [si] and a human face. This way, CMAs of different types were created (e.g., monkey, cow, human, and color). In some cases, the monkeys associated a single sound with several pictures of the same type; for example, four monkey faces were associated with one 'coo,' resulting in four 'monkey' CMAs (****S1 Table****)."* Moreover, S1 Table was tailored to provide better depictions of the different CMAs in our task.

3) CMA acquisition. Do you have acquisition data for every time a new CMA is added? It sounds like once monkey M initially learned a CMA type (the coo, for example), they didn’t take much training to learn to associate a new image with that coo (I believe you added in new individual CMA images gradually, correct? If that’s not the case then I’m misinterpreting the methods and they should be clarified). If they are in fact generalizing that single “coo” learning to new images, that is pretty compelling evidence for a learned label (“coo” means “choose monkey”, for example) rather than many separate individually learned associations. That seems like really relevant information for how these CMAs are acquired and represented, and would definitely be worth adding. You could plot the number of presentations they took to learn each new CMA image, and if they’ve really learned a category label, I would expect those numbers to drop drastically for CMAs acquired later.

Correct, after learning the first CMA, we gradually presented the monkeys with new sound-picture pairs. We added information about learning and performance metrics across sessions in the Methods sections “*Monkeys Training”* and “*Learning Measurements*,” and included Panel B in Fig. 2 (previously S2 Fig), showing the number of sessions in which each CMA was performed above chance after its presentation. However, as the *“Monkeys Training”* section describes, those sessions were counted from the beginning of the last training strategy, which followed many sessions of unsuccessful training strategies. We refer to this at lines 158-177, reading as follows: *“In the subsequent training phase, the monkeys were required to respond to a tone (i.e., a 0.5-second 440Hz; 55 dB SPL) randomly emitted from speakers on either side of the screen. The goal was to indicate the direction of the sound by touching a right or left circle on the screen, which appeared simultaneously with the tone and then after a gradually increasing delay (from 1-3 seconds). Here, the objective was for the monkeys to associate the auditory and visual locations. However, after more than 35,000 trials (i.e., ~ 117 sessions), the performance remained at chance level. Consequently, we adopted a new approach that involved helping the monkeys to directly associate audio cues with specific images.*

*We replaced one circle with a cartoon image of a cow and added a 0.5-second broadband noise, so each trial featured either the tone or the noise. Rewards were given for correctly associating the cow cartoon with the broadband noise and the gray circle with the 440 Hz tone. From then on, sounds were delivered exclusively from a central speaker above the screen, and pictures appeared at different positions but were consistently separated by 180° (of visual angle) from each other. With this new training method, it took only a few sessions for Monkey G to begin performing above chance in associating the broadband noise with the cow cartoon (****S1 Fig****, upper leftmost panel). With many practice sessions, performance improved above chance, prompting us to gradually introduce new sounds and images to establish various CMAs. The initial CMAs involved only two different pictures on the touchscreen, while more complex associations involved the simultaneous presentation of three or four pictures.”*

In this regard, lines 254-265 of the Results explain the following*: “The left panel in* ***Fig 2A*** *illustrates Monkey G's performance for the CMA between the coo sound and the monkey cartoon across sessions. Initially, the performance before learning was at chance level (~300 trials; see methods section on monkeys' training and learning measurements), aligning with the intersection of the learning curve (black line) and the Y-axis, termed Y0. Subsequently, the γ performance level was reached after eight sessions from Y0 (~2700 trials); this level is defined as the session when the HR was above chance, marked by the intersection between the left edge of the gray box and the learning curve. A consistent increase in HR continued until the 15th session, reaching δ performance (right edge of the gray box), and by approximately the 40th session, the performance stabilized at the λ level, where changes in performance from one session to the next were insignificant. Similarly, middle and right panels in* ***Fig 2A*** *show two CMAs learned in trials ending with 3 and 4 pictures, respectively”*

4) Other crossmodal association literature. There is a fair amount of work looking at crossmodal associations in humans and animals, though it tends to be framed more towards identity recognition, that would be worth looking into and including. Here’s a useful review:
Perrodin, C., Kayser, C., Abel, T. J., Logothetis, N. K. & Petkov, C. I. Who is That? Brain Networks and Mechanisms for Identifying Individuals. Trends in Cognitive Sciences 19, 783–796 (2015).

We included information at the Introduction about CMAs in humans, monkeys and even associative learning theories in birds, as well as literature on cross-modal associations at different brain areas. The lines 18-43 read as follows: *“Humans form cross-modal associations (CMAs) between sounds and images, which play a vital role in integrating semantic representations within language [1]. Supporting this, fMRI studies have shown that the temporal lobe of the human brain is actively involved in CMAs [2,3] between words and visual objects [4]. It is believed that CMAs between phonological "templates"—developed in human infants by listening to caretakers—and observed objects are essential for creating semantic representations and aiding the production of a child's first words [5–8]. Similarly, auditory templates have been proposed as a mechanism for vocal production in birds [9–13] and marmoset monkeys [14]. Recent studies, such as those by Carouso-Peck and Goldstein [15,16], have also shown that visual signals during social interactions can also influence vocal production in birds. However, only a few ethological studies have suggested the existence of CMAs between vocal sounds and visual cues for semantic communication [17]. For instance, research has observed that vervet monkeys respond to calls signaling the presence of predators by looking upwards, downwards, or climbing into trees [18].*

*Neurophysiological recordings in monkeys have shown that the prefrontal cortex (PFC) — a brain area homologous to that in humans — utilizes working memory (WM) circuits [19] to perform CMAs between voices and faces [20–32], receiving inputs from various sensory regions [33–36]. CMAs have also been observed in the auditory and visual areas of the temporal lobe [37–45]. Notably, trained macaques have demonstrated the ability to perform cross-modal discriminations between visual and tactile objects [46,47], and between stimuli that could be considered non-ethologically relevant (NER), such as between pitch and color [48] and between amodal information (i.e., information that does not belong to a particular modality) [49] such as numerosity [50] and flutter frequencies [51–54]. However, it remains to be explored whether non-human primates can establish CMAs between NER stimuli that are important for human language, like words —which monkeys can discriminate phonetically [55,56], and pictures.”*

5) Arbitrary learned associations, or existing associations between animals and their calls? Because I was thinking so much about learning, I had a specific running question throughout the paper, which is whether the macaques are actually learning arbitrary associations, or coming in with existing, ecologically relevant associations of certain sounds with certain animals. I think this would be easy to fix with some additional support. If you think they’re arbitrary (and I think I agree with you), you just need to provide evidence. For example, if you have reason to believe the macaques don’t perceive faces on screen as though they’re actual macaques/humans/etc, or that you saw similar patterns even with your cartoon stimuli and the color red, or that they’ve never seen or heard cows before, or something along those lines.

Following the reviewer's concern, we refer only to the CMA between the broadband sound and the cow cartoon as “arbitrary” (S1 Table). However, for the other CMAs, we define them as non-ethologically relevant (NER), being relevant only to humans, such as words and pictures. The evidence presented in the text regarding the NER essence of the stimuli used is described in the results of reaction times (Fig 3), which, together with the time it takes the monkeys to establish CMAs, contrasts with studies showing that associations between ethological stimuli can naturally be observed in monkeys (e.g., Adachi and Hampton 2011).

Specific Suggestions

Abstract:
 Line 1: I would be careful here. Your topic of interest is symbolic crossmodal associations, but even with this cool task, you can’t really make claims that your macaques have learned something symbolic, especially since it’s a unidirectional association and you never test the reverse (i.e. see image choose sound, which would be a bigger methods challenge). Your first line is fine, because it’s true that it is uncertain, but in the second line you need to make it clear that you’re testing for a step below symbolism, just any crossmodal associations at all, and not making any claims about whether they’re represented symbolically in macaques. (see these references for more info on symbolic representations:
Palmer, F. R., & Palmer, F. R. (1981). Semantics. Cambridge University Press.
Deacon, T. W. (1997). The symbolic species: The co-evolution of language and the brain (1st ed). W.W. Norton.)

We agree with the reviewer on the risk of using terms such as "symbolic." We no longer refer to those, but only to associations between complex stimuli. Specifically, lines 1-2 of the Abstract now read as follows: "Humans learn and incorporate cross-modal associations between auditory and visual objects (e.g., between a spoken word and a picture) into language."

Line 6: You say performance remained constant, but I think that undercuts you a bit. They did well, it wasn’t just constant, it was consistently high.

We appreciate this important note. It is correct. Monkeys exhibit consistent high performance while not invariant. Therefore, we no longer refer to perceptual invariance or constancy, but rather we note the monkeys' ability to find equivalences. Consequently, the title of the corresponding Results section is now *"****The Monkeys Recognized Sounds Uttered by Different Speakers****,*" concluding that: *"Altogether, our findings suggest that monkeys can perform CMAs based on the ability to perceive equivalences within different sounds of the same type" (lines 352-354). Similarly, lines 401-404 read as follows: "The animals' performance remained consistently high when the same vocalizations or words were presented with different voices, suggesting that the acoustic variations activated auditory templates, similar to how formants in words trigger acoustic recognition in monkeys [55]."*

Line 9: You say “semantic and conceptual thinking at the single-neuron level,” which doesn’t really say specifically what you mean and also is never mentioned anywhere else in the paper. I would shift this to refer to what you actually conclude they could be a model for, which is, from what I can tell, the neural pathways by which semantic information stored in memory, and later applied.

We agree with the suggestion of reviewer 2, so we replaced the statement with the following (lines 14-16*): “These findings position rhesus monkeys as an ideal model for future research on the brain pathways of cross-modal associations between auditory and visual objects.”*

Intro:
 Line 18: Can you add a reference from human literature to support this claim? It’s not controversial, but there is SO much research on how words are acquired in children, it seems odd to leave it out.

We appreciate this suggestion, we now included references for the Introduction such as Bowerman M, & Choi S. (2001); Vihman M, & Croft W. (2007); Coffey et al (2022), and Bloom & Tinker (2001).

Line 28: See first general suggestion about framing.

We appreciate this suggestion and reframed the manuscript.

Materials and Methods:

Line 77: Stims were presented in an equidistant circle, but did the circle rotate? Was configuration within the circle randomized? How was the actual position and configuration decided?

Lines 118-121 of the methods now state the following: *“After hearing the sound, the animals had to wait during a 3-second delay until 2-4 four pictures were presented simultaneously at random positions but equidistant on an 4° radius from the center of the touchscreen.”*

Line 84: Specify that this is just a cue to denote the start of the trial, I was confused for a moment thinking you were presenting the actual picture before the sound was played.
 Lines 116-118 clarify this as follows: *“Each trial began with a 1° cross appearing in the center of the touchscreen. In response to the cross, the monkeys had to press and hold down a lever so that a 0.5-second reference sound could be delivered.”*

Line 88: This would be a good place to clarify the specifics of the CMA sets.	As mentioned, we improved the description at *the “****Delayed crossmodal match-to-sample task”*** section of the Methods.

Line 100: Here for example, when you say they learned new CMAs, were they all the same type? As in they started out differentiating coos and human words, and the additional CMAs were new images corresponding to coos and words? Or were they totally new CMA types, as in the addition of moos?

We tried to clarify this by referring to CMA types such as ‘monkey’, ‘cow’ and ‘human’, for example, at S1 Table, and lines 125-131 of the methods now read: *“Each CMA was established by associating a sound with a picture representing the same category of external stimulus (e.g., both corresponding to a human). For example, a CMA of the type ‘human’ consisted of the association between the word [si] and a human face. This way, CMAs of different types were created (e.g., monkey, cow, human, and color). In some cases, the monkeys associated a single sound with several pictures of the same type; for example, four monkey faces were associated with one ‘coo’, resulting in four ‘monkey’ CMAs (****S1 Table****).”*

Results:
 Line 119: In S2 Fig B, there are 3 clear dips in performance that I imagine correspond to the addition of a new CMA type/additional foil stimulus, is that right? You should mark where those additions occur on the graph. Unless I’m misinterpreting and this is plotting performance once all 4 CMA types have been added, in which case that should be clarified.

Fig S2 now corresponds to Fig 2. However, we couldn't find an interpretation for those dips, since we averaged the performance at different CMAs at sessions by aligning them all to their first session.

Line 156: They seem to have learned a category set. Coo means touch monkey. So the thing that would slow them down is potentially just how long it takes to recognize that the individual monkeys are monkeys, leading to the reaction time differences (Fig. 2 A and C). When you gave them new images with the sounds, did they generalize to new monkey pictures? This is where the acquisition data would be really useful.

Now Fig 2 is Fig 3. There we had better descriptions about the responses and RTs to the different images. In those cases, there were always trials presenting 3 pictures on screen, so we think it is due to differences between pictures. In Figure 3D, however, it does show, as the reviewer suggests, that the RTs are affected by the number of elements on the screen.

In Fig 2D, it seems the takeaway is that when average RTs are longer their variance is higher, is that correct? So there’s more variance in RTs when they’re taking longer to decide. I wasn’t sure what this graph added until I got to the conclusions, because it seems this pattern is the evidence you’re citing for the visual imagery model. I don’t fully see why this is evidence for that model, so I could use more clarity on that front overall. As it stands I’m not sure that I see the importance of this figure.

We provided of better descriptions to this result by including an improved Fig 3D panel, together with a better wording for its interpretations at lines 332-338, which now read as follows*: “ Furthermore,* ***Fig 3D*** *shows that both the mean and the standard deviation (STD) of the RT distributions increased with the number of pictures displayed on the screen (2-4 pictures), indicating that locating the crossmodal match took longer as the number of distractor pictures increased. This tendency aligns with Weber's Law and studies in time processing [63]. Here, we interpret that the variation in STDs suggests that the faster RTs likely occurred when the matching picture was found first among the pool of pictures on the screen, and longer RTs when the match was found last.”*

Line 172: I would clarify that perceptual invariance is not actually an inability to perceive a difference between the sounds. The critical thing is that they are treating these sounds as equivalent (the way we treat a word as the same no matter whose voice is saying it, or a musical note as the same even if it’s in a different octave), but that they would still likely be able to differentiate the individual sounds if need be. That is an important distinction.

We agree with this interpretation and concluded that*: “regardless of variations in sounds, the animals could recognize them. Altogether, our findings suggest that monkeys can perform CMAs based on the ability to perceive equivalences within different sounds of the same type.”* Lines (351-354)

Possible follow-up thought, I wonder where macaques draw these category lines. Would they view humans saying different words as equivalent, or would different words each have their own set? What about monkey coos vs screams, presumably those would fall into different categories, but could they learn to lump them together into categories of “monkey” and “human” instead?

We don't have much evidence to answer this question, unfortunately. However, anecdotal observations during our experiments suggest that the reviewer is correct.

Discussion:
 Line 191: A point where you could defend that you think these associations are arbitrary.

We no longer use the term “arbitrary” but refer to categories that are not necessarily of ethological relevance (NER), lines (36-38): *“[…] trained macaques have demonstrated the ability to perform cross-modal discriminations between visual and tactile objects [46,47], and between stimuli that could be considered non-ethologically relevant (NER)”*

Line 193: This memory conclusion is surprising, would be less jarring if more theoretical background on this is added to the intro.

We tried to ground the use of WM in our task in the Introduction. In consequence, we also included a WM section in the discussion (“**Working memory mechanisms for crossmodal matching”)** to interpret our results in the scope of established models, and avoided to include our models since we did not provide neurophysiological without evidence. Thus, as said before, we no longer present the former Fig 4 (Fig 4 now is the former Fig3)

Line 220: Do you report this data anywhere?

We left that fact just in sight at Fig 4. We did not discuss it because it was difficult for us to interpret; However, we did not want to remove it either, because we feel that it could provide insights to a reader.

Line 224-230: This explanation helped me understand why you included figure 2, I would try to bring some of this clarity up above.

We included better descriptions in the captions for Fig 4 (previously Fig 3). However, this paragraph is no longer discussed as such. Instead, we focused on providing better descriptions for Fig 3 (previously Fig 2), discussing only that our task demands working memory to transfer information evoked by the sounds (possibly visual cross-modal association) into the comparison period. Specifically, this is addressed in the new "**Working Memory Mechanisms for Crossmodal Matching**" section of the Discussion, to which we referred earlier (lines 418-447).

Line 232: Interesting hypotheses being put forth but again comes out of nowhere, add better framing for it in the intro.

Regardless that we framed the Introduction as suggested by the reviewers, we also followed the reviewer 1’s suggestion in not extending too much in models not supported empirically. So, we avoided interpretations such as “visual imagery”, which no longer appear in the revised version of the manuscript

Line 261: How so? What makes the patten you see a better match for the visual model? Don’t both models require some amount of processing and decision making once the visual stimuli are presented, resulting in differences in RT based on the specifics of the image? Arguably converting an image to a sound to match against your representation of a sound would in fact require more processing at image presentation than matching an image directly to your existing image representation, so wouldn’t the auditory model potentially result in longer and more varied processing once the choice images are presented? Maybe I’m missing something, but it would be useful for you to explain to the readers why you think this model is a better match to your data.

This commentary, together with our response, aligns with the previous concern. In fact, our results can only demonstrate that RT differences relate to the number of pictures rather than the sounds, which, of course, as pointed out by Reviewer 2, does not mean that it was "visual imagery" that was carried in WM. Instead, we only declare that it was information about the sound that was carried in WM (which does not exclude cross-modal information). For example, at lines 419-421, we say: *“In contrast to other tasks [21,37], our* ***monkeys had to retain information about sounds*** *over a 3-second delay and use it to compare with different pictures until they found a match, similar to previous work on the intra- and cross-modal discrimination of flutter [51–54].”*
